# Advancing stem cell technologies for conservation of wildlife biodiversity

**DOI:** 10.1242/dev.203116

**Published:** 2024-10-09

**Authors:** Ashlee M. Hutchinson, Ruth Appeltant, Tom Burdon, Qiuye Bao, Rhishikesh Bargaje, Andrea Bodnar, Stuart Chambers, Pierre Comizzoli, Laura Cook, Yoshinori Endo, Bob Harman, Katsuhiko Hayashi, Thomas Hildebrandt, Marisa L. Korody, Uma Lakshmipathy, Jeanne F. Loring, Clara Munger, Alex H. M. Ng, Ben Novak, Manabu Onuma, Sara Ord, Monique Paris, Andrew J. Pask, Francisco Pelegri, Martin Pera, Ryan Phelan, Benyamin Rosental, Oliver A. Ryder, Woranop Sukparangsi, Gareth Sullivan, Nicole Liling Tay, Nikki Traylor-Knowles, Shawn Walker, Antonia Weberling, Deanne J. Whitworth, Suzannah A. Williams, Jessye Wojtusik, Jun Wu, Qi-Long Ying, Thomas P. Zwaka, Timo N. Kohler

**Affiliations:** ^1^ Revive & Restore, 1505 Bridgeway, Suite 203, Sausalito, CA 94965, USA; ^2^ Gamete Research Centre, Veterinary Physiology and Biochemistry, Department of Veterinary Sciences, University of Antwerp, 2610 Wilrijk, Belgium; ^3^ The Roslin Institute, RDSVS, University of Edinburgh, Easter Bush Campus, Midlothian EH25 9RG, UK; ^4^ IMCB-ESCAR, A*STAR, 61 Biopolis Drive, Proteos, 138673 Singapore; ^5^ Conception Bioscience, Berkeley, CA 94710, USA; ^6^ Gloucester Marine Genomics Institute, 417 Main St, Gloucester, MA 01930, USA; ^7^ Brightfield Therapeutics, South San Francisco, CA 94080, USA; ^8^ Smithsonian National Zoo and Conservation Biology Institute, 3001 Connecticut Ave., NW Washington, DC 20008, USA; ^9^ Lawrence Berkeley National Laboratory, 1 Cyclotron Rd, Berkeley, CA 94720, USA; ^10^ University of California San Diego, 9500 Gilman Dr, La Jolla, CA 92093, USA; ^11^ Vet-Stem Inc. & Personalized Stem Cells, Inc., 14261 Danielson Street, Poway, CA 92064, USA; ^12^ Osaka University, 2-2 Yamadaoka, Suita, Osaka 565-0871, Japan; ^13^ Leibniz Institute for Zoo and Wildlife Research, Alfred-Kowalke-Straße 17, 10315 Berlin, Germany; ^14^ San Diego Zoo Wildlife Alliance, 2920 Zoo Dr, San Diego, CA 92101, USA; ^15^ Thermo Fisher Scientific, 168 Third Avenue, Waltham, MA 02451, USA; ^16^ The Scripps Research Institute, 10550 N Torrey Pines Rd, La Jolla, CA 92037, USA; ^17^ Department of Biochemistry, University of Cambridge, Hopkins Building, Downing Site, Tennis Court Road, Cambridge CB2 1QW, UK; ^18^ GC Therapeutics, 610 Main St., North Cambridge, MA 02139, USA; ^19^ National Institute for Environmental Studies, 16-2 Onogawa, City of Tsukuba, Ibaraki 305-8506, Japan; ^20^ Colossal Biosciences, 1401 Lavaca St, Unit #155 Austin, TX 78701, USA; ^21^ IBREAM (Institute for Breeding Rare and Endangered African Mammals), Edinburgh EH3 6AT, UK; ^22^ University of Melbourne, Parkville, VIC 3052, Australia; ^23^ University of Wisconsin-Madison, 500 Lincoln Dr, Madison, WI 53706, USA; ^24^ Jackson Laboratory, 600 Main Street, Bar Harbor, ME 04609, USA; ^25^ The Shraga Segal Department of Microbiology, Immunology, and Genetics, Faculty of Health Sciences, Center for Regenerative Medicine and Stem Cells, Ben Gurion University of the Negev, Beer Sheva 8410501, Israel; ^26^ Department of Biology, Faculty of Science, Burapha University, 169 Long-Had Bangsaen Rd, Saen Suk, Chon Buri District, Chon Buri 20131, Thailand; ^27^ Department of Pediatric Research, Oslo University Hospital, P.O. Box 4950 Nydalen, N-0424 Oslo, Norway; ^28^ School of Medicine, University of St Andrews, North Haugh, St Andrews KY16 9TF, UK; ^29^ Rosenstiel School of Marine, Atmospheric, and Earth Science, University of Miami, 4600, Rickenbacker Cswy, Key Biscayne, FL 33149, USA; ^30^ ViaGen Pets & Equine, PO Box 1119, Cedar Park, TX 78613, USA; ^31^ All Souls College, University of Oxford, Oxford OX1 4AL, UK; ^32^ University of Queensland, Sir Fred Schonell Drive, Brisbane, Queensland, 4072, Australia; ^33^ University of Oxford, Oxford OX1 2JD, UK; ^34^ Omaha's Henry Doorly Zoo & Aquarium, 3701 S 10th St, Omaha, NE 68107, USA; ^35^ University of Texas Southwestern Medical Center, 5323 Harry Hines Blvd, Dallas, TX 75390, USA; ^36^ Keck School of Medicine of University of Southern California, 1975 Zonal Ave, Los Angeles, CA 90033, USA; ^37^ Department of Cell, Developmental, and Regenerative Biology, and Black Family Stem Cell Institute, Icahn School of Medicine at Mount Sinai, New York, NY 10029, USA

**Keywords:** Biodiversity, Conservation, Disease modelling, *In vitro* gametogenesis, Stem cells, IPSC

## Abstract

Wildlife biodiversity is essential for healthy, resilient and sustainable ecosystems. For biologists, this diversity also represents a treasure trove of genetic, molecular and developmental mechanisms that deepen our understanding of the origins and rules of life. However, the rapid decline in biodiversity reported recently foreshadows a potentially catastrophic collapse of many important ecosystems and the associated irreversible loss of many forms of life on our planet. Immediate action by conservationists of all stripes is required to avert this disaster. In this Spotlight, we draw together insights and proposals discussed at a recent workshop hosted by Revive & Restore, which gathered experts to discuss how stem cell technologies can support traditional conservation techniques and help protect animal biodiversity. We discuss reprogramming, *in vitro* gametogenesis, disease modelling and embryo modelling, and we highlight the prospects for leveraging stem cell technologies beyond mammalian species.

## Teaser

**Summary:** Species loss is occurring at unprecedented speed, outpacing traditional conservation measures. This Spotlight explores the potential for stem cell technologies to support genetic rescue and conservation.

## Introduction

**Fig. 1.**
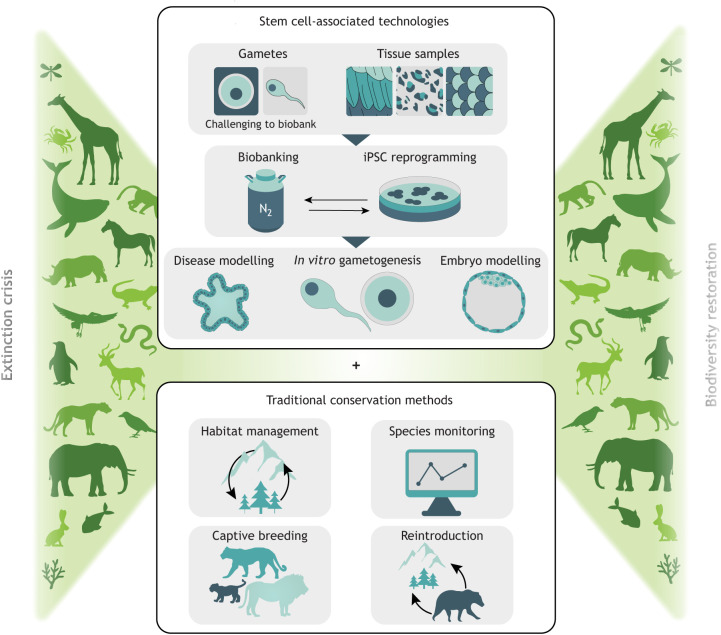
A synergistic approach to wildlife conservation combining stem cell-associated technologies and traditional conservation methods. Induced pluripotent stem cell (iPSC) reprogramming allows efficient biobanking for conservation and supports technologies such as *in vitro* gametogenesis, disease and embryo modelling. Reproductive material such as gametes and embryos can be difficult to obtain for endangered species, as well as challenging to cryopreserve with high post-thaw viability. Easily cryopreserved, pluripotent stem cells could be used to produce germ cells and contribute to embryo formation. Moreover, pluripotent stem cells are an expandable resource, with rapid proliferation and unlimited self-renewal. In contrast, primary cell lines represent a limited supply incapable of prolonged culture. Owing to their capacity to become disease-pertinent cell types, iPSCs additionally offer a downstream resource for the study and treatment of disease and will be essential tools for bioengineering resilience. Establishing reprogramming protocols now rather than later will be important for identifying problems with sampling, donor and cell type for select species. These stem cell technologies will complement traditional methods such as habitat management, species monitoring, captive breeding and reintroduction (Moloney et al., 2023; Sutherland et al., 2021). Deriving disease-resistant embryos for select endangered species is unlikely to result in recovery for that species without established and suitable habitat. However, habitat management alone may not work fast enough to protect critically endangered species that require reproductive support. Together, these methods can address the extinction crisis driven by human-induced biodiversity loss and promote biodiversity restoration.

We are currently witnessing the sixth mass extinction event for life on Earth, posing unprecedented challenges for conservation biology. In contrast to previous extinction events, human-driven species losses are occurring exceptionally rapidly. Extinctions are estimated to be hundreds or thousands of times higher than expected background rates and have the potential to irrevocably alter the biosphere (Ceballos and Ehrlich, 2023). The scale and pace of this event demands concerted action from all areas of conservation biology to curb a catastrophic loss of biodiversity (Ceballos and Ehrlich, 2023). We propose that stem cell-associated techniques and their potential to develop new avenues for assisted reproductive technologies (ART) can complement traditional conservation approaches (such as habitat restoration and species monitoring; Fig. 1) and may play an important role in countering the effects of this extinction crisis (Hildebrandt et al., 2021; Saragusty et al., 2016). Pluripotent stem cells (PSCs) are a promising addition to the conservation toolkit, with the potential to become any cell type within an organism. PSCs can be derived directly from embryos or by converting somatic cells to induced pluripotent stem cells (iPSCs) (Evans and Kaufman, 1981; Martin, 1981; Takahashi and Yamanaka, 2006). Obtaining embryos for PSC generation is challenging for most mammalian species (Bolton et al., 2022), but iPSCs offer an alternative way to harness the same developmental potential for multiple target species and are suggested to be functionally equivalent to embryo-derived PSCs (Yamanaka, 2012).

Since their first derivation in 2006, iPSCs have been recognised for their potential to transform the fields of regenerative medicine, disease modelling and reproduction (Takahashi and Yamanaka, 2006). They also offer promise for protecting our planet's wildlife, as reprogramming technology offers a way to transform primary cells into an unlimited resource with a wide array of downstream applications such as *in vitro* gametogenesis and disease and embryo modelling. These applications could be leveraged to support traditional conservation techniques (Fig. 1) (Mooney et al., 2023). For example, biobanks are currently freezing both gametes and primary cell lines to safeguard the genetic diversity of potentially all (endangered) species for future biodiversity restoration efforts (Ballou et al., 2023; Bolton et al., 2022). However, cryopreserved cell lines are limited in utility, and reproductive material is challenging to obtain and preserve (Hildebrandt et al., 2021). *In vitro* gametogenesis could complement these biobanking efforts, as the ability to produce germ cells and embryos from biobank samples, independent of individual animals, would increase recovery options in extreme endangerment cases, such as for the northern white rhinoceros (*Ceratotherium simum cottoni*). Importantly, iPSCs also offer a route to modified offspring, delivering loss- and gain-of-function models, essential for functional genomics and performing facilitated adaptation (Thomas et al., 2013). Optimised, standardised and accessible reprogramming protocols applicable to a range of species will mark a new era in applied biobanking, providing the means to store valuable genomic information in a pluripotent form as stem cell ‘zoos’ (Lázaro et al., 2023).

The first iPSCs from endangered species were reported thirteen years ago (Ben-Nun et al., 2011), and subsequent studies have expanded this approach to other endangered animals (Ben-Nun et al., 2011; Endo et al., 2022; Hildebrandt et al., 2018; Honda et al., 2017; Katayama et al., 2022; Ramaswamy et al., 2015; Sukparangsi et al., 2022; Verma et al., 2012, 2013; Weeratunga et al., 2018; Whitworth et al., 2019; Zywitza et al., 2022). However, there has been only slow progress toward integrating stem cell technologies within conservation. To address this challenge, the recent Stem Cell Technology for Genetic Rescue workshop, held in La Jolla, California in September 2023 and hosted by Revive & Restore, brought together scientists from diverse fields with the aim of deepening understanding surrounding species-specificities in pluripotency and differentiation. Participants explored the capacity for iPSC technologies to support traditional conservation efforts, highlighting the involvement of stem cell scientists as valuable contributors to the development of genetic rescue strategies for endangered species. In this Spotlight, we discuss the core focus areas selected during the workshop for their capacity to support applied biobanking and animal biodiversity restoration: reprogramming, *in vitro* gametogenesis, and disease and embryo modelling. Although we do not discuss plants here, stem cell technology also has the potential to support the conservation of plant species with seeds that cannot be preserved or for which cell culture is challenging (Greb and Lohmann, 2016). We call on the scientific community to prioritise the development of improved, reproducible and more accessible protocols for stem cell and associated technologies for the conservation of biodiversity.

## Generating pluripotent stem cells in endangered species

### Reprogramming across diverse taxa

Research into iPSC derivation has primarily focused on species with clinical, evolutionary or agricultural significance. For example, bat iPSCs were recently used to explore tolerance for high viral load with implications for COVID-19 (Déjosez et al., 2023), primate iPSCs are used as tools to unpack human evolution (Gallego Romero et al., 2015) and attempts to optimise the challenging process of bovine reprogramming have persisted largely due to the agricultural relevance of cattle (Déjosez et al., 2023; Gallego Romero et al., 2015; Pillai et al., 2021). Although there has been a gradual increase in the reported derivation of pluripotent cells from endangered species, these efforts cannot access funding reserved for biomedical applications and usually proceed no further than proof-of-concept demonstrations.

Although the core regulatory network for maintaining pluripotency, including the transcription factors OCT3/4, SOX2 and NANOG, is well-documented in vertebrates (Endo et al., 2020), differences in signalling pathways and isoforms across species underscore the need for further exploration (Fu et al., 2018; Kumar et al., 2022). In addition, when aiming to establish defined states of pluripotency, such as naive (pre-implantation) and primed (post-implantation), distinct signalling requirements become apparent (Marks et al., 2012; Nichols and Smith, 2009). In mouse PSCs, WNT signalling promotes naive pluripotency, whereas inhibition of WNT signalling supports human naive pluripotency (Bredenkamp et al., 2019; Ying et al., 2008). As a result, optimising reprogramming protocols can be labour-intensive, with each species requiring adjustments in methodology. For example, felid reprogramming is enhanced by the addition of NANOG (Verma et al., 2012), whereas successful platypus iPSC production has typically involved the presence of leukaemia inhibitory factor, basic fibroblast growth factor and a range of inhibitors targeting the MEK, ALK, GSKβ and TGFβ pathways (Whitworth et al., 2019). Variable results have been reported for hypoxic conditions in cattle and rabbits (Bessi et al., 2021; Honda et al., 2010) and the use of knock-out serum replacement (KOSR) instead of foetal bovine serum (FBS) promotes rhesus monkey reprogramming (Liu et al., 2008). Some species present additional challenges, appearing to be resistant to reprogramming (Kuzma-Hunt et al., 2023; Pillai et al., 2019). This may be because of epigenetic barriers, as reported for the naked mole rat (Tan et al., 2017), or multiple copies of the tumour suppressor gene *p53*, as is the case in the elephant (Appleton et al., 2024 preprint)*.* Here, the SV40 large T-antigen was used to modulate p53 levels (Appleton et al., 2024 preprint). Overexpression of the SV40 large T-antigen has also been used to overcome reprogramming barriers observed for goat and sheep by dramatically increasing proliferation (Bao et al., 2011; Mali et al., 2008; Ren et al., 2011). Substantial variation in gene expression, as well as the capacity for chimerism and germline contribution, has been observed for derived iPSCs across species (Lee et al., 2017; West et al., 2011); however, because of methodological differences, it is unclear whether these discrepancies originate in inconsistent benchmarking, species-specific variation or protocol alterations. Standardised validation and improved understanding of pluripotency state transitions, as well as refining and manipulating culture conditions, will be key to producing high-quality iPSCs across taxa that can be used for conservation.

The delivery method for the reprogramming factors must also be considered. Non-integrative vectors, such as Sendai virus, mRNA or the reprogramming factor proteins themselves, maintain genomic integrity and are suitable for applications in biodiversity conservation (Nishimura et al., 2011; Okita et al., 2011). When reprogramming fails, species-specific transcription factors offer an alternative to using human or mouse factors (Appleton et al., 2024 preprint; Liu et al., 2008). Owing to their unlimited self-renewal capacity, iPSC cultures are prone to accumulating genetic mutations, so the maintenance of genomic integrity is both challenging and essential (Endo et al., 2022; Koh et al., 2013; Park et al., 2015). Regular monitoring can be performed using available methods, including karyotyping, genome sequencing and chromosome mapping, which currently represent the most effective approaches for detecting these aberrations (Ludwig et al., 2023). However, even iPSCs harbouring proliferation-associated mutations can still be valuable as research tools, offering insights into differentiation pathways and disease mechanisms, and serving as a repository for the genetic diversity of species (Lee et al., 2017; Noto et al., 2014; Song et al., 2021).

Although many challenges remain, we note that reprogramming efforts for conservation would greatly benefit from expanded access to cell lines and insights gained from emerging fields such as cellular agriculture, alongside enhanced resources for endangered species and optimised derivation processes (Box 1).

**Box 1. Broader access and industry intersections**

The use of iPSCs allows storage of multiple samples, enabling a decentralised network and allowing local communities to hold their own biobanks. This will become increasingly important, as the Nagoya protocol places renewed emphasis on indigenous and local sovereignty (Beato and Veneroso, 2023).

Ensuring easier global access to cell lines, currently limited by legal frameworks (Karesh et al., 2016), along with enhanced in-country expertise, will support improved reprogramming across species that will both enable and be supported by robust comparisons of pluripotency. However, the expense of stem cell derivation and maintenance poses a challenge for under-resourced nations within biodiversity hotspots. Cheaper alternatives to stem cell media and growth factors would enhance local capacity. Moreover, education and training, along with accessible protocols, are needed to elucidate stem cell processes for conservation scientists, veterinary staff and the zoo community. Therefore, collaborations between academia, the biobanking community and industry groups with a stake in stem cell research for non-model species must be nurtured.

Conventionally viewed as a non-profitable area, stem cell technology for diverse species is now converging with industry directions for the first time. Research and development initiatives for lab-grown meat and textiles, longevity and human gamete production are emerging sectors that will benefit from an expanded understanding of stem cell induction, regulation and differentiation. Similarly, the veterinary industry stands to gain from improved protocols for stem cell derivation in different species, as well as opportunities for new treatments.

### Validation of pluripotent stem cell lines

After generating PSC lines, it is crucial to validate them as such using standardised benchmarking. This can be achieved by assessing different criteria such as morphology, self-renewal, gene expression levels, protein levels, methylation states and silencing of ectopic reprogramming factors (Boroviak and Nichols, 2017; Ying and Smith, 2017). However, the ultimate test of pluripotency is to demonstrate germline transmission by creating chimaeras (incorporating PSCs into an embryo of another individual) that contain PSC-derived germ cells, which is not possible without established reproductive technologies and access to embryos (Bradley et al., 1984; Okita, 2007). Although key features of pluripotency have been extensively characterised in humans and rodents (Du and Wu, 2024; Smith, 2001), the validation of pluripotency in non-model species remains challenging. This is largely due to a lack of species-specific antibodies, availability of reference genomes (Wang et al., 2021 preprint) and challenges in obtaining embryos for comparison.

Conventional assays for validating pluripotency may not be available for endangered species, making it essential to establish realistic benchmarking standards. These standards should include evidence of differentiation into all three germ layers, as outlined by the ISSCR guidelines (https://www.isscr.org/standards-document). One approach is the transplantation of putative PSCs into immunodeficient mice to assess teratoma formation, which would confirm differentiation into endodermal, mesodermal and ectodermal derivatives (Evans and Kaufman, 1981; Martin, 1981). Alternatively, *in vitro* tri-lineage differentiation can be demonstrated using embryoid body assays, where PSCs are cultured in suspension to form spherical aggregates that differentiate into various cell types (Desbaillets et al., 2000; Doetschman et al., 1985). The use of transcriptomic atlases may also provide a valuable tool for characterising pluripotency and validating differentiation outcomes (Malkowska et al., 2022). Finally, interspecies chimera technology offers an alternative route for *in vivo* differentiation and germline transmission tracking (Wu et al., 2017). This method involves integrating PSCs from endangered species into embryos of more readily available model organisms, potentially overcoming the limitations of traditional validation approaches.

## Applications of stem cell technologies in endangered species

### *In vitro* gametogenesis

*In vitro* gametogenesis (IVG) involves generating spermatozoa or oocytes outside of a living organism (Saitou and Hayashi, 2021), paving the way for assisted reproductive technologies such as *in vitro* fertilisation (IVF) or intracytoplasmic sperm injection (ICSI) to induce fertilisation and produce embryos. The latest innovations in murine IVG research might offer solutions for the most hopeless situations where genetic material may only be available for one sex, as XY chromosomes can now be converted into XX in pluripotent stem cells and deployed for *in vitro* oogenesis (Murakami et al., 2023). Complete IVG has been achieved only in mice but it does provide proof-of-concept for using this approach in other species (Hayashi et al., 2012; Saitou and Hayashi, 2021; Yoshino et al., 2021). Unlike embryo modelling or cloning, this technology enables sexual reproduction and recombination, producing new genetic profiles (Cowl et al., 2024). This is an important advantage because dwindling populations experience reduced genetic diversity.

The derivation of gametes from iPSCs is an integral part of the strategy to save the northern white rhinoceros (Hayashi et al., 2022; Hildebrandt et al., 2021; Korody et al., 2021; Saragusty et al., 2016). This project is ongoing, but has already established primordial germ cell-like cells (PGCLCs) from northern white rhinoceros iPSCs (Hayashi et al., 2022; Korody et al., 2021). However, a solid understanding of the reproductive physiology of any target species will be fundamental to achieving healthy live births (Comizzoli and Holt, 2019; Herrick, 2019; Mastromonaco and Songsasen, 2020). Although the closely related southern white rhinoceros can provide such information for the northern white rhinoceros, not every species has a readily available close relative. Bridging the gap between the promising results obtained in mice and IVG for endangered species will require optimisation in large domestic animal models such as pigs or cattle, as well as non-human primates (Gyobu-Motani et al., 2023; Seita et al., 2023). This approach also requires a robust understanding of the molecular pathways that regulate the generation of germ and supporting cells (such as granulosa and Sertoli cells) *in vitro*. Initial IVG efforts relied on access to primary supporting cells, but recent work demonstrates the feasibility of deriving these directly from iPSCs (Yoshino et al., 2021).

Notably, IVG technology could also be used to generate spermatogonial stem and progenitor cells (SSPCs) for use in conservation biology. In mice and livestock (pigs, goats and sheep) SSPCs can be transplanted into the testes of recipient males (Ciccarelli et al., 2020; Zhao et al., 2021a) and these can then be returned to the population to spread new or lost diversity. This approach could also be used to introduce genetic modifications into endangered animals without requiring full IVG protocols or ART for every species.

As iPSC technology continues to advance, IVG provides a promising way to ensure the reproductive viability of threatened species. While these advanced artificial reproductive techniques are developed, biobanking provides a buffer across time to store not only genomic information, but also precious cellular material (Hildebrandt et al., 2021). Although cryopreservation of germ cells such as spermatozoa and oocytes would facilitate immediate fertilisation, biobanks often focus on collection of tissue samples and cell lines because of practical and technical limitations of cryopreservation methods (Bolton et al., 2022; Hildebrandt et al., 2021). IVG will play a key role in using these somatic tissues for reproductive purposes. However, the use of stem cell-associated reproductive technologies poses challenges, so these approaches should be initiated early and in parallel with enhanced cryopreservation techniques for continued banking of reproductive material. For example, germ cells derived from iPSCs have not been regulated by germline protective mechanisms and are more vulnerable to mutation (Saitou and Hayashi, 2021), so their (epi)genetic quality must be examined closely. Indeed, offspring born from these sources may exhibit genetic abnormalities and replicating the epigenetic state of primordial germ cells *in vitro* remains challenging (Bhartiya et al., 2014). Leveraging precursors of the endogenous pre-existing stem cells within the ovary (oogonial stem cells) could help address this caveat, as these cells express specific markers and exhibit the epigenetic profile of primordial germ cells (Bhartiya et al., 2014). In mice, these ovarian stem cells survive oncotherapy, differentiate into oocyte-like structures and result in healthy offspring (Zou et al., 2009). However, iPSC technology remains crucial in scenarios where ovarian tissue is absent.

### Disease modelling

In addition to their potential for generating gametes *in vitro*, iPSCs can serve as an unlimited source of differentiated somatic cell types for deployment in developing effective monitoring and mitigating strategies for disease, toxins and other environmental challenges.

Understanding barriers to disease transmission is essential for protecting vulnerable populations. iPSCs from susceptible wild species could provide relevant cell types to understand the basis of disease resilience and susceptibility and develop potential therapeutic or prophylactic measures. This might prove particularly useful in safeguarding wild bird populations from avian flu, wild dogs and carnivores from distemper, or wild pigs from African Swine Fever Virus. iPSCs could also generate elements of the immune system and pathogen-targeted tissues to develop culture and three-dimensional (3D) organoid experimental systems that more accurately model disease phenotypes (Sharma et al., 2020). For example, horse iPSC-derived neurons have been used to investigate susceptibility to neurotropic Flavivirus infection (Fortuna et al., 2018), and pig PSC-derived macrophages present new opportunities to investigate resilience to pathogens such as African Swine Fever Virus that threaten both domestic pig and endangered wild pig populations (Meek et al., 2022). Fine-tuning PSC differentiation protocols to generate phenotypically relevant cells at scale will be a major future challenge in maximising the utility of these disease studies. This will involve translating and optimising existing protocols, as well as the generation of new methods.

In the context of long-term changes in global temperatures and weather patterns, understanding resilience against environmental change is also crucial. A pioneering study, comparing human and hibernating ground squirrel iPSC-derived neurons, identified key biochemical stress pathways that, when modulated appropriately, improved resistance to thermal stress in iPSC-derived neurons from both humans and rats (Ou et al., 2018). Understanding mechanisms underpinning resilience is particularly important for developing strategies aimed at protecting the keystone species in threatened communities such as corals, which we discuss in more detail below.

### Embryo modelling

The exceptional ability of PSCs to organise themselves into complex structures has driven significant advancements in creating 3D structures known as stem cell-based embryo models (SCBEMs) that replicate various early mammalian developmental stages, from pre-implantation through to the beginning of organ formation (Wu and Fu, 2024). These exhibit varying degrees of resemblance to actual embryos in terms of shape, overall gene expression profiles and cellular composition (Wu and Fu, 2024). One of the most promising applications of SCBEMs for species preservation is the creation of pre-implantation blastocyst models, called ‘blastoids’ (Oura et al., 2023), for reproductive purposes, which will be the focus of discussion here. Notably, SCBEMs offer the potential for genetic rescue and broader biodiversity conservation efforts through the generation of reproductive cells; however, these applications will not be discussed here.

In recent years, blastoid models have been created across a variety of mammalian species, including mice (Li et al., 2019; Rivron et al., 2018; Sozen et al., 2019), humans (Kagawa et al., 2021; Yanagida et al., 2021; Yu et al., 2021), cattle (Pinzón-Arteaga et al., 2023), pigs (Xiang et al., 2024), monkeys (Li et al., 2023) and bats (Déjosez et al., 2023). These models effectively replicate the essential cell types needed for both the development of the foetus and the tissues that support it, such as the trophectoderm and hypoblast. Blastoids are produced through various methods: they can be formed by guiding a single type of embryo-derived PSC to generate both the embryo and the support tissues (Li et al., 2019; Yu et al., 2021), by mixing embryo-derived PSCs with cells destined to become support tissues (Pinzón-Arteaga et al., 2023; Rivron et al., 2018) or through reprogramming of somatic cells to make iPSCs that can be used as a starting population (Liu et al., 2021). Murine (Li et al., 2019; Rivron et al., 2018), monkey (Li et al., 2023) and bovine (Pinzón-Arteaga et al., 2023) blastoids placed into surrogate mothers can initiate early stages of pregnancy. However, blastoids transferred to the uterus have not yet developed sufficiently to result in the birth of offspring. As implantation might be the bottleneck, *in vitro* platforms could facilitate improvements at the endometrial-blastoid interface (Shibata et al., 2024). To date, no blastoids have been developed for endangered species, representing an unexplored area of potential. To move closer to this objective, the field must advance our knowledge of early development in different species, establishing more effective embryo cultures (Aguilera-Castrejon et al., 2021) and PSC conditions (Du and Wu, 2024), as well as refining reprogramming methods (MacCarthy et al., 2024). Such advancements are crucial for harnessing the full potential of SCBEM technology.

## Beyond mammalian stem cells

The extensive groundwork already done in mice and humans, along with the availability of ARTs, suggests that implementing stem cell approaches is most achievable for mammalian endangered species. However, recent advancements in PSC research are expanding the possibilities across a broader range of taxa. The following sections discuss the potential of stem cell technologies beyond mammals, including avian species, non-avian reptiles and amphibians, and marine invertebrate species.

### Avian species

About 12% of avian species are currently threatened with extinction (www.iucnredlist.org). Both embryo-derived PSCs and iPSCs have been obtained for avian species, exhibiting similarities in gene regulatory networks to mammals (Intarapat and Stern, 2013). Avian species present an advantageous system for embryonic integration as stem cells can be injected directly into the embryo within the egg to generate chimaeras (Intarapat and Stern, 2013). Recently, iPSCs from four endangered avian species were generated using standard reprogramming factors, plus KLF2 and YAP (Katayama et al., 2022). Although the derived cells expressed core pluripotency factors including *POU5F1*, *LIN28A/B* and *NANOG*, gene expression and pathway analysis differed from the standard murine profile. SOX3 was more highly expressed than SOX2, highlighting its active role in avian pluripotency (Whitworth et al., 2019). iPSCs derived from the Japanese ptarmigan could integrate into chicken embryos and produce interspecific chimeras, although germline competence was not observed (Katayama et al., 2022). Developing protocols for germline-competent avian PSCs would be a significant breakthrough in avian transgenesis, holding immense promise for conservation. Differentiation of avian iPSCs to primordial germ cells *in vitro*, followed by embryo-injection via the egg to complete gametogenesis *in vivo*, will address avian-specific challenges for performing IVF by facilitating natural reproduction. However, the germline-restricted chromosome in songbirds poses a challenge for using somatic cells as a starting point (Borodin, 2023).

### Non-avian reptiles and amphibians

Reptile and amphibian populations are experiencing sharp declines worldwide (Strand et al., 2020). Although iPSC technology has not yet been reported for non-avian reptiles, stem cell-derived organoids from snakes represent advances for this taxon (Post et al., 2020). As numerous reptiles exhibit temperature-dependent developmental and physiological processes, the derivation of stem cells could offer an avenue to explore the impact of climate change on these species. Similarly, although amphibian reprogramming remains unexplored, it holds the potential to facilitate disease modelling, particularly in response to the deadly chytrid fungus (Bolton et al., 2022). iPSC cultures might improve amphibian cell yield and utility for downstream research (Strand et al., 2020). Interestingly, intramuscular injection of the Yamanaka factors in tadpoles results in upregulation of core pluripotency markers, suggesting conservation of Yamanaka-induced reprogramming for this taxon (Vivien et al., 2012).

### Marine invertebrate species

Marine invertebrates represent a substantial portion of global biodiversity (Bodnar, 2016; Chen, 2021). Currently, reef-building corals are under severe threat from increasing ocean temperatures that lead to bleaching. Because coral responses to stress vary (Palumbi et al., 2014; van Oppen et al., 2018), one conservation goal has been imparting stress-resilient genotypes (National Academies of Sciences, Engineering, and Medicine, 2019). Transferring genetic properties from one coral to another or, after manipulation, back to the same coral, requires the ability to isolate stem-like progenitor cells and engraft them through transplantation. Preliminary work suggests that candidate stem cells in the sea anemone *Nematostella vectensis* can proliferate and integrate, contributing to gene and phenotype transfer, cell differentiation and longevity, for genetic rescue (Talice et al., 2023). Many marine invertebrates exhibit indeterminate growth, high regenerative capacity and asexual modes of reproduction, suggesting robust stem cell-like properties. However, little is known about their stem cell biology (Ballarin, Rinkevich and Hobmayer, 2022). The ability to culture stem cells from marine invertebrates would provide a powerful resource for understanding their biology, symbiosis, disease aetiology and stress response, and could even provide an alternative to wild harvest through cellular agriculture (Rubio et al., 2019). A collaborative effort is needed to develop an integrated systems-level approach to optimise *in vitro* culture conditions, devise markers to validate cell identity and prioritise taxa for which cell culture tools can address the most pressing problems facing marine ecosystems.

## Perspective

Translating iPSC technology to wildlife conservation might provide a way to both safeguard and produce resilient individuals from endangered animal species. It is, therefore, paramount to fund and develop parallel methods for germ cell derivation, as well as apply clinical stem cell research to wildlife disease. Learning from non-mammalian species and harnessing their developmental potential will deliver essential insight into the effects of the climate crisis, as well as provide solutions to protect the Earth's biodiversity.

The Stem Cell Technology for Genetic Rescue 2023 workshop aimed to accelerate advancements and foster collaborations in this rapidly evolving field. Workshop participants identified key barriers, including a lack of funding and the fragmentation of research within the conservation community. In contrast to the frequent convening of experts in biomedicine, stem cell researchers working on endangered species often operate in isolation due to the lack of dedicated scientific meetings. Enhancing research transparency and fostering cross-disciplinary knowledge sharing is essential to expedite progress and prevent redundancy.

Currently, the literature prioritises close alignment of results with the mouse model, limiting the exploration of pluripotency as a biological property and an evolutionary feature. Standardising both reprogramming methods and approaches to benchmarking will enable accurate comparisons across taxa. For example, there is a clear need to improve and align transcriptomic resources for PSC gene regulatory networks across species to deepen our understanding of pluripotency. This may include standardising RNA sequencing methods (Ramsköld et al., 2012) and will depend on generating robust reference genomes for more species. Comparing expression profiles across proven pluripotent stem cells from a wide diversity of species could be used to define broadly conserved networks associated with pluripotency (Déjosez et al., 2023; Kumar et al., 2022; Whitworth et al., 2019). Open access to transcriptomic profiles for reprogrammed cells across species will be crucial for expanding knowledge of pluripotency regulation across evolutionary time, and integrating this information as a landscape for stem cell states in vertebrates is a first vital step toward developing a more universal reprogramming toolkit. In future, this technology could also be applied to more diverged groups, such as invertebrates.

Stem cell technology has the capacity to augment traditional conservation efforts, as biobanking and advanced ART offer emergency measures to conserve both species and genetic material. However, their impact and measurable effects on conservation remain to be determined (Sutherland et al., 2021). Therefore, these technologies and approaches should be integrated with and funded alongside established conservation approaches. As habitat restoration and climate action struggle to keep pace with rapid species decline, stem cell-associated techniques offer an additional buffer to mitigate extinctions. Leveraging this potential will complement current conservation efforts to safeguard species diversity. Ultimately, however, as with any conservation measure, continued protection of suitable habitats for wildlife will be essential for maintaining a healthy, resilient and biodiverse planet.

## References

[DEV203116C1] Aguilera-Castrejon, A., Oldak, B., Shani, T., Ghanem, N., Itzkovich, C., Slomovich, S., Tarazi, S., Bayerl, J., Chugaeva, V., Ayyash, M. et al. (2021). Ex utero mouse embryogenesis from pre-gastrulation to late organogenesis. *Nature* 593, 119-124. 10.1038/s41586-021-03416-3 33731940

[DEV203116C2] Appleton, E., Hong, K., Rodriguez, C., Tanaka, Y., Ashkenazy-Titelman, A., Bhide, K., Rasmussen-Ivey, C., Ambriz-Pena, X., Korover, N., Bai, H. et al. (2024). Derivation of elephant induced pluripotent stem cells. *bioRxiv* 10.1101/2024.03.05.583606 42332088

[DEV203116C3] Ballarin, L., Rinkevich, B., Hobmayer, B. eds. (2022). *Advances in aquatic invertebrate stem cell research: from basic research to innovative applications*. Basel, Switzerland: MDPI.

[DEV203116C4] Ballou, J. D., Lacy, R. C., Traylor-Holzer, K., Bauman, K., Ivy, J. A. and Asa, C. (2023). Strategies for establishing and using genome resource banks to protect genetic diversity in conservation breeding programs. *Zoo Biol.* 42, 175-184. 10.1002/zoo.21741 36205245

[DEV203116C5] Bao, L., He, L., Chen, J., Wu, Z., Liao, J., Rao, L., Ren, J., Li, H., Zhu, H., Qian, L. et al. (2011). Reprogramming of ovine adult fibroblasts to pluripotency via drug-inducible expression of defined factors. *Cell Res.* 21, 600-608. 10.1038/cr.2011.6 21221129 PMC3203662

[DEV203116C6] Beato, M. S. and Veneroso, V. (2023). The Nagoya Protocol on access and benefit sharing: the neglected issue of animal health. *Front. Microbiol.* 14, 1124120. 10.3389/fmicb.2023.1124120 36865778 PMC9971722

[DEV203116C7] Ben-Nun, I. F., Montague, S. C., Houck, M. L., Tran, H. T., Garitaonandia, I., Leonardo, T. R., Wang, Y.-C., Charter, S. J., Laurent, L. C., Ryder, O. A. et al. (2011). Induced pluripotent stem cells from highly endangered species. *Nat. Methods* 8, 829-831. 10.1038/nmeth.1706 21892153

[DEV203116C8] Bessi, B. W., Botigelli, R. C., Pieri, N. C. G., Machado, L. S., Cruz, J. B., de Moraes, P., de Souza, A. F., Recchia, K., Barbosa, G., de Castro, R. V. G. et al. (2021). Cattle in vitro induced pluripotent stem cells generated and maintained in 5 or 20% oxygen and different supplementation. *Cells* 10, 1531. 10.3390/cells10061531 34204517 PMC8234940

[DEV203116C9] Bhartiya, D., Hinduja, I., Patel, H. and Bhilawadikar, R. (2014). Making gametes from pluripotent stem cells--a promising role for very small embryonic-like stem cells. *Reprod. Biol. Endocrinol.* 12, 114. 10.1186/1477-7827-12-114 25421462 PMC4255929

[DEV203116C10] Bodnar, A. (2016). Lessons from the sea: marine animals provide models for biomedical research. *Environment* 58, 16-25. 10.1080/00139157.2016.1134020

[DEV203116C11] Bolton, R. L., Mooney, A., Pettit, M. T., Bolton, A. E., Morgan, L., Drake, G. J., Appeltant, R., Walker, S. L., Gillis, J. D. and Hvilsom, C. (2022). Resurrecting biodiversity: advanced assisted reproductive technologies and biobanking. *Reprod. Fertil.* 3, R121-R146. 10.1530/RAF-22-0005 35928671 PMC9346332

[DEV203116C12] Borodin, P. M. (2023). Germline-restricted chromosomes of the songbirds. *Vavilovskii Zhurnal Genet. Selektsii* 27, 641-650. 10.18699/VJGB-23-75 38023808 PMC10643108

[DEV203116C13] Boroviak, T. and Nichols, J. (2017). Primate embryogenesis predicts the hallmarks of human naïve pluripotency. *Development* 144, 175-186. 10.1242/dev.145177 28096211 PMC5430762

[DEV203116C14] Bradley, A., Evans, M., Kaufman, M. H. and Robertson, E. (1984). Formation of germ-line chimaeras from embryo-derived teratocarcinoma cell lines. *Nature* 309, 255-256. 10.1038/309255a0 6717601

[DEV203116C15] Bredenkamp, N., Yang, J., Clarke, J., Stirparo, G. G., von Meyenn, F., Dietmann, S., Baker, D., Drummond, R., Ren, Y., Li, D. et al. (2019). Wnt inhibition facilitates RNA-mediated reprogramming of human somatic cells to naive pluripotency. *Stem Cell Reports* 13, 1083-1098. 10.1016/j.stemcr.2019.10.009 31708477 PMC6915845

[DEV203116C16] Ceballos, G. and Ehrlich, P. R. (2023). Mutilation of the tree of life via mass extinction of animal genera. *Proc. Natl. Acad. Sci. USA* 120, e2306987120. 10.1073/pnas.2306987120 37722053 PMC10523489

[DEV203116C17] Chen, E. Y.-S. (2021). Often overlooked: understanding and meeting the current challenges of marine invertebrate conservation. *Front. Mar. Sci.* 8, 690704. 10.3389/fmars.2021.690704

[DEV203116C18] Ciccarelli, M., Giassetti, M. I., Miao, D., Oatley, M. J., Robbins, C., Lopez-Biladeau, B., Waqas, M. S., Tibary, A., Whitelaw, B., Lillico, S. et al. (2020). Donor-derived spermatogenesis following stem cell transplantation in sterile NANOS2 knockout males. *Proc. Natl. Acad. Sci. USA* 117, 24195-24204. 10.1073/pnas.2010102117 32929012 PMC7533891

[DEV203116C19] Comizzoli, P. and Holt, W. V. (2019). Breakthroughs and new horizons in reproductive biology of rare and endangered animal species. *Biol. Reprod.* 101, 514-525. 10.1093/biolre/ioz031 30772911

[DEV203116C20] Cowl, V. B., Comizzoli, P., Appeltant, R., Bolton, R. L., Browne, R. K., Holt, W. V., Penfold, L. M., Swegen, A., Walker, S. L. and Williams, S. A. (2024). Cloning for the twenty-first century and its place in endangered species conservation. *Annu. Rev. Anim. Biosci.* 12, 91-112. 10.1146/annurev-animal-071423-093523 37988633

[DEV203116C21] Déjosez, M., Marin, A., Hughes, G. M., Morales, A. E., Godoy-Parejo, C., Gray, J. L., Qin, Y., Singh, A. A., Xu, H., Juste, J. et al. (2023). Bat pluripotent stem cells reveal unusual entanglement between host and viruses. *Cell* 186, 957-974.e28. 10.1016/j.cell.2023.01.011 36812912 PMC10085545

[DEV203116C22] Desbaillets, I., Ziegler, U., Groscurth, P. and Gassmann, M. (2000). Embryoid bodies: an in vitro model of mouse embryogenesis. *Exp. Physiol.* 85, 645-651. 10.1111/j.1469-445X.2000.02104.x 11187960

[DEV203116C23] Doetschman, T. C., Eistetter, H., Katz, M., Schmidt, W. and Kemler, R. (1985). The in vitro development of blastocyst-derived embryonic stem cell lines: formation of visceral yolk sac, blood islands and myocardium. *J. Embryol. Exp. Morphol.* 87, 27-45. 10.1242/dev.87.1.27 3897439

[DEV203116C24] Du, P. and Wu, J. (2024). Hallmarks of totipotent and pluripotent stem cell states. *Cell Stem Cell* 31, 312-333. 10.1016/j.stem.2024.01.009 38382531 PMC10939785

[DEV203116C25] Endo, Y., Kamei, K.-I. and Inoue-Murayama, M. (2020). Genetic signatures of evolution of the pluripotency gene regulating network across mammals. *Genome Biol. Evol.* 12, 1806-1818. 10.1093/gbe/evaa169 32780791 PMC7643368

[DEV203116C26] Endo, Y., Kamei, K.-I., Hasegawa, K., Okita, K., Ito, H., Terada, S. and Inoue-Murayama, M. (2022). Generation and gene expression profiles of Grevy's zebra induced pluripotent stem cells. *Stem Cells Dev.* 31, 250-257. 10.1089/scd.2021.0253 35316100

[DEV203116C27] Evans, M. J. and Kaufman, M. H. (1981). Establishment in culture of pluripotential cells from mouse embryos. *Nature* 292, 154-156. 10.1038/292154a0 7242681

[DEV203116C28] Fortuna, P. R. J., Bielefeldt-Ohmann, H., Ovchinnikov, D. A., Wolvetang, E. J. and Whitworth, D. J. (2018). Cortical neurons derived from equine induced pluripotent stem cells are susceptible to neurotropic Flavivirus infection and replication: an in vitro model for equine neuropathic diseases. *Stem Cells Dev.* 27, 704-715. 10.1089/scd.2017.0106 29562867

[DEV203116C29] Fu, K., Chronis, C., Soufi, A., Bonora, G., Edwards, M., Smale, S. T., Zaret, K. S., Plath, K. and Pellegrini, M. (2018). Comparison of reprogramming factor targets reveals both species-specific and conserved mechanisms in early iPSC reprogramming. *BMC Genomics* 19, 956. 10.1186/s12864-018-5326-1 30577748 PMC6303873

[DEV203116C31] Gallego Romero, I., Pavlovic, B. J., Hernando-Herraez, I., Zhou, X., Ward, M. C., Banovich, N. E., Kagan, C. L., Burnett, J. E., Huang, C. H., Mitrano, A. et al. (2015). A panel of induced pluripotent stem cells from chimpanzees: a resource for comparative functional genomics. *eLife* 4, e07103. 10.7554/eLife.07103 26102527 PMC4502404

[DEV203116C32] Greb, T. and Lohmann, J. U. (2016). Plant stem cells. *Curr. Biol.* 26, R816-R821. 10.1016/j.cub.2016.07.070 27623267

[DEV203116C33] Gyobu-Motani, S., Yabuta, Y., Mizuta, K., Katou, Y., Okamoto, I., Kawasaki, M., Kitamura, A., Tsukiyama, T., Iwatani, C., Tsuchiya, H. et al. (2023). Induction of fetal meiotic oocytes from embryonic stem cells in cynomolgus monkeys. *EMBO J.* 42, e112962. 10.15252/embj.2022112962 36929479 PMC10152148

[DEV203116C34] Hayashi, K., Ogushi, S., Kurimoto, K., Shimamoto, S., Ohta, H. and Saitou, M. (2012). Offspring from oocytes derived from in vitro primordial germ cell–like cells in mice. *Science* 338, 971-975. 10.1126/science.1226889 23042295

[DEV203116C35] Hayashi, M., Zywitza, V., Naitou, Y., Hamazaki, N., Goeritz, F., Hermes, R., Holtze, S., Lazzari, G., Galli, C., Stejskal, J. et al. (2022). Robust induction of primordial germ cells of white rhinoceros on the brink of extinction. *Sci. Adv.* 8, eabp9683. 10.1126/sciadv.abp9683 36490332 PMC9733929

[DEV203116C36] Herrick, J. R. (2019). Assisted reproductive technologies for endangered species conservation: developing sophisticated protocols with limited access to animals with unique reproductive mechanisms. *Biol. Reprod.* 100, 1158-1170. 10.1093/biolre/ioz025 30770538

[DEV203116C37] Hildebrandt, T. B., Hermes, R., Colleoni, S., Diecke, S., Holtze, S., Renfree, M. B., Stejskal, J., Hayashi, K., Drukker, M., Loi, P. et al. (2018). Embryos and embryonic stem cells from the white rhinoceros. *Nat. Commun.* 9, 2589. 10.1038/s41467-018-04959-2 29973581 PMC6031672

[DEV203116C38] Hildebrandt, T. B., Hermes, R., Goeritz, F., Appeltant, R., Colleoni, S., de Mori, B., Diecke, S., Drukker, M., Galli, C., Hayashi, K. et al. (2021). The ART of bringing extinction to a freeze - History and future of species conservation, exemplified by rhinos. *Theriogenology* 169, 76-88. 10.1016/j.theriogenology.2021.04.006 33940218

[DEV203116C39] Honda, A., Hirose, M., Hatori, M., Matoba, S., Miyoshi, H., Inoue, K. and Ogura, A. (2010). Generation of induced pluripotent stem cells in rabbits. *J. Biol. Chem.* 285, 31362-31369. 10.1074/jbc.M110.150540 20670936 PMC2951210

[DEV203116C40] Honda, A., Choijookhuu, N., Izu, H., Kawano, Y., Inokuchi, M., Honsho, K., Lee, A.-R., Nabekura, H., Ohta, H., Tsukiyama, T. et al. (2017). Flexible adaptation of male germ cells from female iPSCs of endangered *Tokudaia osimensis*. *Sci. Adv.* 3, e1602179. 10.1126/sciadv.1602179 28508054 PMC5429033

[DEV203116C41] Intarapat, S. and Stern, C. D. (2013). Chick stem cells: current progress and future prospects. *Stem Cell Res.* 11, 1378-1392. 10.1016/j.scr.2013.09.005 24103496 PMC3989061

[DEV203116C42] Kagawa, H., Javali, A., Khoei, H. H., Sommer, T. M., Sestini, G., Novatchkova, M., Scholte op Reimer, Y., Castel, G., Bruneau, A., Maenhoudt, N. et al. (2021). Human blastoids model blastocyst development and implantation. *Nature* 601, 600-605. 10.1038/s41586-021-04267-8 34856602 PMC8791832

[DEV203116C43] Karesh, W. B., Kock, R. and Machalaba, C. C. (2016). CITES: in sickness and in health? *Ecohealth* 13, 441-442. 10.1007/s10393-016-1154-4 27541636

[DEV203116C44] Katayama, M., Fukuda, T., Kaneko, T., Nakagawa, Y., Tajima, A., Naito, M., Ohmaki, H., Endo, D., Asano, M., Nagamine, T. et al. (2022). Induced pluripotent stem cells of endangered avian species. *Commun. Biol.* 5, 1049. 10.1038/s42003-022-03964-y 36280684 PMC9592614

[DEV203116C45] Koh, S., Thomas, R., Tsai, S., Bischoff, S., Lim, J.-H., Breen, M., Olby, N. J. and Piedrahita, J. A. (2013). Growth requirements and chromosomal instability of induced pluripotent stem cells generated from adult canine fibroblasts. *Stem Cells Dev.* 22, 951-963. 10.1089/scd.2012.0393 23016947 PMC3585736

[DEV203116C46] Korody, M. L., Ford, S. M., Nguyen, T. D., Pivaroff, C. G., Valiente-Alandi, I., Peterson, S. E., Ryder, O. A. and Loring, J. F. (2021). Rewinding extinction in the northern white rhinoceros: genetically diverse induced pluripotent stem cell bank for genetic rescue. *Stem Cells Dev.* 30, 177-189. 10.1089/scd.2021.0001 33406994 PMC7891310

[DEV203116C47] Kumar, S., De Leon, E. M., Granados, J., Whitworth, D. J. and VandeBerg, J. L. (2022). Monodelphis domestica induced pluripotent stem cells reveal metatherian pluripotency architecture. *Int. J. Mol. Sci.* 23, 12623. 10.3390/ijms232012623 36293487 PMC9604385

[DEV203116C48] Kuzma-Hunt, A. G., Shah, V., DiMarco, S., Russell, K. A., Betts, D. H. and Koch, T. G. (2023). Opening the “black box” underlying barriers to the use of canine induced pluripotent stem cells: a narrative review. *Stem Cells Dev.* 32, 271-291. 10.1089/scd.2022.0300 36884307 PMC10278003

[DEV203116C150] Lázaro, J., Costanzo, M., Sanaki-Matsumiya, M., Girardot, C., Hayashi, M., Hayashi, K., Diecke, S., Hildebrandt, T. B., Lazzari, G., Wu, J. et al. (2023). A stem cell zoo uncovers intracellular scaling of developmental tempo across mammals. *Cell Stem Cell* 30, 938-949.e7. 10.1016/j.stem.2023.05.014 37343565 PMC10321541

[DEV203116C49] Lee, S.-G., Mikhalchenko, A. E., Yim, S. H., Lobanov, A. V., Park, J.-K., Choi, K.-H., Bronson, R. T., Lee, C.-K., Park, T. J. and Gladyshev, V. N. (2017). Naked mole rat induced pluripotent stem cells and their contribution to interspecific chimera. *Stem Cell Rep.* 9, 1706-1720. 10.1016/j.stemcr.2017.09.013 PMC5829328 29107591

[DEV203116C50] Li, R., Zhong, C., Yu, Y., Liu, H., Sakurai, M., Yu, L., Min, Z., Shi, L., Wei, Y., Takahashi, Y. et al. (2019). Generation of blastocyst-like structures from mouse embryonic and adult cell cultures. *Cell* 179, 687-702.e18. 10.1016/j.cell.2019.09.029 31626770 PMC7359735

[DEV203116C51] Li, J., Zhu, Q., Cao, J., Liu, Y., Lu, Y., Sun, Y., Li, Q., Huang, Y., Shang, S., Bian, X. et al. (2023). Cynomolgus monkey embryo model captures gastrulation and early pregnancy. *Cell Stem Cell* 30, 362-377.e7. 10.1016/j.stem.2023.03.009 37028403

[DEV203116C52] Liu, H., Zhu, F., Yong, J., Zhang, P., Hou, P., Li, H., Jiang, W., Cai, J., Liu, M., Cui, K. et al. (2008). Generation of induced pluripotent stem cells from adult rhesus monkey fibroblasts. *Cell Stem Cell* 3, 587-590. 10.1016/j.stem.2008.10.014 19041774

[DEV203116C53] Liu, X., Tan, J. P., Schröder, J., Aberkane, A., Ouyang, J. F., Mohenska, M., Lim, S. M., Sun, Y. B. Y., Chen, J., Sun, G. et al. (2021). Modelling human blastocysts by reprogramming fibroblasts into iBlastoids. *Nature* 591, 627-632. 10.1038/s41586-021-03372-y 33731926

[DEV203116C54] Ludwig, T. E., Andrews, P. W., Barbaric, I., Benvenisty, N., Bhattacharyya, A., Crook, J. M., Daheron, L. M., Draper, J. S., Healy, L. E., Huch, M. et al. (2023). ISSCR standards for the use of human stem cells in basic research. *Stem Cell Rep.* 18, 1744-1752. 10.1016/j.stemcr.2023.08.003 PMC10545481 37703820

[DEV203116C55] MacCarthy, C. M., Wu, G., Malik, V., Menuchin-Lasowski, Y., Velychko, T., Keshet, G., Fan, R., Bedzhov, I., Church, G. M., Jauch, R. et al. (2024). Highly cooperative chimeric super-SOX induces naive pluripotency across species. *Cell Stem Cell* 31, 127-147.e9. 10.1016/j.stem.2023.11.010 38141611

[DEV203116C56] Mali, P., Ye, Z., Hommond, H. H., Yu, X., Lin, J., Chen, G., Zou, J. and Cheng, L. (2008). Improved efficiency and pace of generating induced pluripotent stem cells from human adult and fetal fibroblasts. *Stem Cells* 26, 1998-2005. 10.1634/stemcells.2008-0346 18511599

[DEV203116C57] Malkowska, A., Penfold, C., Bergmann, S. and Boroviak, T. E. (2022). A hexa-species transcriptome atlas of mammalian embryogenesis delineates metabolic regulation across three different implantation modes. *Nat. Commun.* 13, 3407. 10.1038/s41467-022-30194-x 35710749 PMC9203550

[DEV203116C58] Marks, H., Kalkan, T., Menafra, R., Denissov, S., Jones, K., Hofemeister, H., Nichols, J., Kranz, A., Stewart, A. F., Smith, A. et al. (2012). The transcriptional and epigenomic foundations of ground state pluripotency. *Cell* 149, 590-604. 10.1016/j.cell.2012.03.026 22541430 PMC3398752

[DEV203116C59] Martin, G. R. (1981). Isolation of a pluripotent cell line from early mouse embryos cultured in medium conditioned by teratocarcinoma stem cells. *Proc. Natl. Acad. Sci. USA* 78, 7634-7638. 10.1073/pnas.78.12.7634 6950406 PMC349323

[DEV203116C60] Mastromonaco, G. F. and Songsasen, N. (2020). Reproductive technologies for the conservation of wildlife and endangered species. In *Reproductive Technologies in Animals* (ed. G. A. Presicce), pp. 99-117. Elsevier.

[DEV203116C61] Meek, S., Watson, T., Eory, L., McFarlane, G., Wynne, F. J., McCleary, S., Dunn, L. E. M., Charlton, E. M., Craig, C., Shih, B. et al. (2022). Stem cell-derived porcine macrophages as a new platform for studying host-pathogen interactions. *BMC Biol.* 20, 14. 10.1186/s12915-021-01217-8 35027054 PMC8759257

[DEV203116C62] Moloney, D. J. F., Collins, C., Holloway, P. and O'Riordan, R. (2023). The Conservationist's Toolkit: a critical review of the need for a conceptual framework of both *in-situ* and ex-situ conservation strategies to ensure the success of restoration ecology. *Biol. Conserv.* 287, 110345. 10.1016/j.biocon.2023.110345

[DEV203116C63] Mooney, A., Ryder, O. A., Houck, M. L., Staerk, J., Conde, D. A. and Buckley, Y. M. (2023). Maximizing the potential for living cell banks to contribute to global conservation priorities. *Zoo Biol.* 42, 697-708. 10.1002/zoo.21787 37283210

[DEV203116C64] Murakami, K., Hamazaki, N., Hamada, N., Nagamatsu, G., Okamoto, I., Ohta, H., Nosaka, Y., Ishikura, Y., Kitajima, T. S., Semba, Y. et al. (2023). Generation of functional oocytes from male mice in vitro. *Nature* 615, 900-906. 10.1038/s41586-023-05834-x 36922585

[DEV203116C65] National Academies of Sciences, Engineering, and Medicine (2019). *A Research Review of Interventions to Increase the Persistence and Resilience of Coral Reefs*. Washington, D.C: National Academies Press.

[DEV203116C66] Nichols, J. and Smith, A. (2009). Naive and primed pluripotent states. *Cell Stem Cell* 4, 487-492. 10.1016/j.stem.2009.05.015 19497275

[DEV203116C67] Nishimura, K., Sano, M., Ohtaka, M., Furuta, B., Umemura, Y., Nakajima, Y., Ikehara, Y., Kobayashi, T., Segawa, H., Takayasu, S. et al. (2011). Development of defective and persistent Sendai virus vector. *J. Biol. Chem.* 286, 4760-4771. 10.1074/jbc.M110.183780 21138846 PMC3039346

[DEV203116C68] Noto, F. K., Determan, M. R., Cai, J., Cayo, M. A., Mallanna, S. K. and Duncan, S. A. (2014). Aneuploidy is permissive for hepatocyte-like cell differentiation from human induced pluripotent stem cells. *BMC Res. Notes* 7, 437. 10.1186/1756-0500-7-437 25002137 PMC4105394

[DEV203116C69] Okita, K. (2007). Generation of germline-competent induced pluripotent stem cells. *Nature* 448, 313-317. 10.1038/nature05934 17554338

[DEV203116C70] Okita, K., Matsumura, Y., Sato, Y., Okada, A., Morizane, A., Okamoto, S., Hong, H., Nakagawa, M., Tanabe, K., Tezuka, K.-I. et al. (2011). A more efficient method to generate integration-free human iPS cells. *Nat. Methods* 8, 409-412. 10.1038/nmeth.1591 21460823

[DEV203116C71] Ou, J., Ball, J. M., Luan, Y., Zhao, T., Miyagishima, K. J., Xu, Y., Zhou, H., Chen, J., Merriman, D. K., Xie, Z. et al. (2018). IPSCs from a hibernator provide a platform for studying cold adaptation and its potential medical applications. *Cell* 173, 851-863.e16. 10.1016/j.cell.2018.03.010 29576452 PMC5935596

[DEV203116C72] Oura, S., Hamilton, J. N. and Wu, J. (2023). Recent advances in stem cell-based blastocyst models. *Curr. Opin. Genet. Dev.* 81, 102088. 10.1016/j.gde.2023.102088 37451164 PMC12077647

[DEV203116C73] Palumbi, S. R., Barshis, D. J., Traylor-Knowles, N. and Bay, R. A. (2014). Mechanisms of reef coral resistance to future climate change. *Science* 344, 895-898. 10.1126/science.1251336 24762535

[DEV203116C74] Park, H.-S., Hwang, I., Choi, K.-A., Jeong, H., Lee, J.-Y. and Hong, S. (2015). Generation of induced pluripotent stem cells without genetic defects by small molecules. *Biomaterials* 39, 47-58. 10.1016/j.biomaterials.2014.10.055 25477171

[DEV203116C75] Pillai, V. V., Kei, T. G., Reddy, S. E., Das, M., Abratte, C., Cheong, S. H. and Selvaraj, V. (2019). Induced pluripotent stem cell generation from bovine somatic cells indicates unmet needs for pluripotency sustenance. *Anim. Sci. J.* 90, 1149-1160. 10.1111/asj.13272 31322312

[DEV203116C76] Pillai, V. V., Koganti, P. P., Kei, T. G., Gurung, S., Butler, W. R. and Selvaraj, V. (2021). Efficient induction and sustenance of pluripotent stem cells from bovine somatic cells. *Biol. Open* 10, bio058756. 10.1242/bio.058756 34719702 PMC8565620

[DEV203116C77] Pinzón-Arteaga, C. A., Wang, Y., Wei, Y., Ribeiro Orsi, A. E., Li, L., Scatolin, G., Liu, L., Sakurai, M., Ye, J., Ming, H. et al. (2023). Bovine blastocyst-like structures derived from stem cell cultures. *Cell Stem Cell* 30, 611-616.e7. 10.1016/j.stem.2023.04.003 37146582 PMC10230549

[DEV203116C78] Post, Y., Puschhof, J., Beumer, J., Kerkkamp, H. M., de Bakker, M. A. G., Slagboom, J., de Barbanson, B., Wevers, N. R., Spijkers, X. M., Olivier, T. et al. (2020). Snake venom gland organoids. *Cell* 180, 233-247.e21. 10.1016/j.cell.2019.11.038 31978343

[DEV203116C79] Ramaswamy, K., Yik, W. Y., Wang, X.-M., Oliphant, E. N., Lu, W., Shibata, D., Ryder, O. A. and Hacia, J. G. (2015). Derivation of induced pluripotent stem cells from orangutan skin fibroblasts. *BMC Res. Notes* 8, 577. 10.1186/s13104-015-1567-0 26475477 PMC4609060

[DEV203116C80] Ramsköld, D., Luo, S., Wang, Y.-C., Li, R., Deng, Q., Faridani, O. R., Daniels, G. A., Khrebtukova, I., Loring, J. F., Laurent, L. C. et al. (2012). Full-length mRNA-Seq from single-cell levels of RNA and individual circulating tumor cells. *Nat. Biotechnol.* 30, 777-782. 10.1038/nbt.2282 22820318 PMC3467340

[DEV203116C81] Ren, J., Pak, Y., He, L., Qian, L., Gu, Y., Li, H., Rao, L., Liao, J., Cui, C., Xu, X. et al. (2011). Generation of hircine-induced pluripotent stem cells by somatic cell reprogramming. *Cell Res.* 21, 849-853. 10.1038/cr.2011.37 21403680 PMC3203672

[DEV203116C82] Rivron, N. C., Frias-Aldeguer, J., Vrij, E. J., Boisset, J.-C., Korving, J., Vivié, J., Truckenmüller, R. K., van Oudenaarden, A., van Blitterswijk, C. A. and Geijsen, N. (2018). Blastocyst-like structures generated solely from stem cells. *Nature* 557, 106-111. 10.1038/s41586-018-0051-0 29720634

[DEV203116C83] Rubio, N., Datar, I., Stachura, D., Kaplan, D. and Krueger, K. (2019). Cell-based fish: a novel approach to seafood production and an opportunity for cellular agriculture. *Front. Sustain. Food Syst.* 3, 43. 10.3389/fsufs.2019.00043

[DEV203116C84] Saitou, M. and Hayashi, K. (2021). Mammalian in vitro gametogenesis. *Science* 374, aaz6830. 10.1126/science.aaz6830 34591639

[DEV203116C85] Saragusty, J., Diecke, S., Drukker, M., Durrant, B., Friedrich Ben-Nun, I., Galli, C., Göritz, F., Hayashi, K., Hermes, R., Holtze, S. et al. (2016). Rewinding the process of mammalian extinction. *Zoo Biol.* 35, 280-292. 10.1002/zoo.21284 27142508

[DEV203116C86] Seita, Y., Cheng, K., McCarrey, J. R., Yadu, N., Cheeseman, I. H., Bagwell, A., Ross, C. N., Santana Toro, I., Yen, L.-H., Vargas, S. et al. (2023). Efficient generation of marmoset primordial germ cell-like cells using induced pluripotent stem cells. *eLife* 12, e82263. 10.7554/eLife.82263 36719274 PMC9937652

[DEV203116C87] Sharma, A., Sances, S., Workman, M. J. and Svendsen, C. N. (2020). Multi-lineage human iPSC-derived platforms for disease modeling and drug discovery. *Cell Stem Cell* 26, 309-329. 10.1016/j.stem.2020.02.011 32142662 PMC7159985

[DEV203116C88] Shibata, S., Endo, S., Nagai, L. A. E., Kobayashi, E. H., Oike, A., Kobayashi, N., Kitamura, A., Hori, T., Nashimoto, Y., Nakato, R. et al. (2024). Modeling embryo-endometrial interface recapitulating human embryo implantation. *Sci. Adv.* 10, eadi4819. 10.1126/sciadv.adi4819 38394208 PMC10889356

[DEV203116C89] Smith, A. G. (2001). Embryo-derived stem cells: of mice and men. *Annu. Rev. Cell Dev. Biol.* 17, 435-462. 10.1146/annurev.cellbio.17.1.435 11687496

[DEV203116C90] Song, J. H. T., Grant, R. L., Behrens, V. C., Kučka, M., Roberts Kingman, G. A., Soltys, V., Chan, Y. F. and Kingsley, D. M. (2021). Genetic studies of human–chimpanzee divergence using stem cell fusions. *Proc. Natl. Acad. Sci. USA* 118, e2117557118. 10.1073/pnas.2117557118 34921118 PMC8713981

[DEV203116C91] Sozen, B., Cox, A. L., De Jonghe, J., Bao, M., Hollfelder, F., Glover, D. M. and Zernicka-Goetz, M. (2019). Self-organization of mouse stem cells into an extended potential blastoid. *Dev. Cell* 51, 698-712.e8. 10.1016/j.devcel.2019.11.014 31846649 PMC10291877

[DEV203116C92] Strand, J., Thomsen, H., Jensen, J. B., Marcussen, C., Nicolajsen, T. B., Skriver, M. B., Søgaard, I. M., Ezaz, T., Purup, S., Callesen, H. et al. (2020). Biobanking in amphibian and reptilian conservation and management: opportunities and challenges. *Conserv. Genet. Resour.* 12, 709-725. 10.1007/s12686-020-01142-y

[DEV203116C93] Sukparangsi, W., Thongphakdee, A., Karoon, S., Suban Na Ayuthaya, N., Hengkhunthod, I., Prakongkaew, R., Bootsri, R. and Sikaeo, W. (2022). Establishment of fishing cat cell biobanking for sustainable conservation. *Front. Vet. Sci.* 9, 989670. 10.3389/fvets.2022.989670 36439340 PMC9684188

[DEV203116C94] Sutherland, W. J., Dicks, L. V., Petrovan, S. O. and Smith, R. K. eds. (2021). *What Works in Conservation 2021*. Open Book Publishers.

[DEV203116C95] Takahashi, K. and Yamanaka, S. (2006). Induction of pluripotent stem cells from mouse embryonic and adult fibroblast cultures by defined factors. *Cell* 126, 663-676. 10.1016/j.cell.2006.07.024 16904174

[DEV203116C96] Talice, S., Barkan, S. K., Snyder, G. A., Ottolenghi, A., Eliachar, S., Ben-Romano, R., Oisher, S., Sharoni, T., Lewandowska, M., Sultan, E. et al. (2023). Candidate stem cell isolation and transplantation in Hexacorallia. *Dev Comp Immunol* 148, 105012. 10.1016/j.dci.2023.105012 PMC12715556 39487989

[DEV203116C97] Tan, L., Ke, Z., Tombline, G., Macoretta, N., Hayes, K., Tian, X., Lv, R., Ablaeva, J., Gilbert, M., Bhanu, N. V. et al. (2017). Naked mole rat cells have a stable epigenome that resists iPSC reprogramming. *Stem Cell Rep.* 9, 1721-1734. 10.1016/j.stemcr.2017.10.001 PMC5831052 29107597

[DEV203116C98] Thomas, M. A., Roemer, G. W., Donlan, C. J., Dickson, B. G., Matocq, M. and Malaney, J. (2013). Ecology: gene tweaking for conservation. *Nature* 501, 485-486. 10.1038/501485a 24073449

[DEV203116C99] van Oppen, M. J. H., Bongaerts, P., Frade, P., Peplow, L. M., Boyd, S. E., Nim, H. T. and Bay, L. K. (2018). Adaptation to reef habitats through selection on the coral animal and its associated microbiome. *Mol. Ecol.* 27, 2956-2971. 10.1111/mec.14763 29900626

[DEV203116C100] Verma, R., Holland, M. K., Temple-Smith, P. and Verma, P. J. (2012). Inducing pluripotency in somatic cells from the snow leopard (Panthera uncia), an endangered felid. *Theriogenology* 77, 220-228.e2. 10.1016/j.theriogenology.2011.09.022 22079579

[DEV203116C101] Verma, R., Liu, J., Holland, M. K., Temple-Smith, P., Williamson, M. and Verma, P. J. (2013). Nanog is an essential factor for induction of pluripotency in somatic cells from endangered felids. *Biores. Open Access* 2, 72-76. 10.1089/biores.2012.0297 23514873 PMC3569963

[DEV203116C102] Vivien, C., Scerbo, P., Girardot, F., Le Blay, K., Demeneix, B. A. and Coen, L. (2012). Non-viral expression of mouse Oct4, Sox2, and Klf4 transcription factors efficiently reprograms tadpole muscle fibers in vivo. *J. Biol. Chem.* 287, 7427-7435. 10.1074/jbc.M111.324368 22232554 PMC3293547

[DEV203116C103] Wang, G., Brändl, B., Rohrandt, C., Hong, K., Pang, A., Lee, J., Lewin, H. A., Migliorelli, G., Stanke, M., Schwab, R. et al. (2021). Chromosome-level genome assembly of the functionally extinct northern white rhinoceros (*Ceratotherium simum cottoni*). *bioRxiv* 10.1101/2021.12.11.472206 PMC12107126 40359041

[DEV203116C104] Weeratunga, P., Shahsavari, A., Ovchinnikov, D. A., Wolvetang, E. J. and Whitworth, D. J. (2018). Induced pluripotent stem cells from a marsupial, the Tasmanian devil (Sarcophilus harrisii): insight into the evolution of mammalian pluripotency. *Stem Cells Dev.* 27, 112-122. 10.1089/scd.2017.0224 29161957

[DEV203116C105] West, F. D., Uhl, E. W., Liu, Y., Stowe, H., Lu, Y., Yu, P., Gallegos-Cardenas, A., Pratt, S. L. and Stice, S. L. (2011). Brief report: chimeric pigs produced from induced pluripotent stem cells demonstrate germline transmission and no evidence of tumor formation in young pigs. *Stem Cells* 29, 1640-1643. 10.1002/stem.713 22039609

[DEV203116C106] Whitworth, D. J., Limnios, I. J., Gauthier, M.-E., Weeratunga, P., Ovchinnikov, D. A., Baillie, G., Grimmond, S. M., Graves, J. A. M. and Wolvetang, E. J. (2019). Platypus induced pluripotent stem cells: the unique pluripotency signature of a monotreme. *Stem Cells Dev.* 28, 151-164. 10.1089/scd.2018.0179 30417748

[DEV203116C118] Wu, J. and Fu, J. (2024). Toward developing human organs via embryo models and chimeras. *Cell* 187, 3194-3219. 10.1016/j.cell.2024.05.027 38906095 PMC11239105

[DEV203116C107] Wu, J., Platero-Luengo, A., Sakurai, M., Sugawara, A., Gil, M. A., Yamauchi, T., Suzuki, K., Bogliotti, Y. S., Cuello, C., Morales Valencia, M. et al. (2017). Interspecies chimerism with mammalian pluripotent stem cells. *Cell* 168, 473-486.e15. 10.1016/j.cell.2016.12.036 28129541 PMC5679265

[DEV203116C119] Xiang, J., Wang, H., Shi, B., Li, J., Liu, D., Wang, K., Wang, Z., Min, Q., Zhao, C. and Pei, D. (2024). Pig blastocyst-like structure models from embryonic stem cells. *Cell Discov*. 10, 72. 10.1038/s41421-024-00693-w 38956027 PMC11219778

[DEV203116C108] Yamanaka, S. (2012). Induced pluripotent stem cells: past, present, and future. *Cell Stem Cell* 10, 678-684. 10.1016/j.stem.2012.05.005 22704507

[DEV203116C109] Yanagida, A., Spindlow, D., Nichols, J., Dattani, A., Smith, A. and Guo, G. (2021). Naive stem cell blastocyst model captures human embryo lineage segregation. *Cell Stem Cell* 28, 1016-1022.e4. 10.1016/j.stem.2021.04.031 33957081 PMC8189436

[DEV203116C110] Ying, Q.-L. and Smith, A. (2017). The art of capturing pluripotency: creating the right culture. *Stem Cell Rep.* 8, 1457-1464. 10.1016/j.stemcr.2017.05.020 PMC5470336 28591647

[DEV203116C111] Ying, Q.-L., Wray, J., Nichols, J., Batlle-Morera, L., Doble, B., Woodgett, J., Cohen, P. and Smith, A. (2008). The ground state of embryonic stem cell self-renewal. *Nature* 453, 519-523. 10.1038/nature06968 18497825 PMC5328678

[DEV203116C112] Yoshino, T., Suzuki, T., Nagamatsu, G., Yabukami, H., Ikegaya, M., Kishima, M., Kita, H., Imamura, T., Nakashima, K., Nishinakamura, R. et al. (2021). Generation of ovarian follicles from mouse pluripotent stem cells. *Science* 373, eabe0237. 10.1126/science.abe0237 34437124

[DEV203116C113] Yu, L., Wei, Y., Duan, J., Schmitz, D. A., Sakurai, M., Wang, L., Wang, K., Zhao, S., Hon, G. C. and Wu, J. (2021). Blastocyst-like structures generated from human pluripotent stem cells. *Nature* 591, 620-626. 10.1038/s41586-021-03356-y 33731924

[DEV203116C114] Zhao, X., Wan, W., Zhang, X., Wu, Z. and Yang, H. (2021a). Spermatogonial stem cell transplantation in large animals. *Animals (Basel)* 11, 918. 10.3390/ani11040918 33805058 PMC8064064

[DEV203116C116] Zou, K., Yuan, Z., Yang, Z., Luo, H., Sun, K., Zhou, L., Xiang, J., Shi, L., Yu, Q., Zhang, Y. et al. (2009). Production of offspring from a germline stem cell line derived from neonatal ovaries. *Nat. Cell Biol.* 11, 631-636. 10.1038/ncb1869 19363485

[DEV203116C117] Zywitza, V., Frahm, S., Krüger, N., Weise, A., Göritz, F., Hermes, R., Holtze, S., Colleoni, S., Galli, C., Drukker, M. et al. (2022). Induced pluripotent stem cells and cerebral organoids from the critically endangered Sumatran rhinoceros. *iScience* 25, 105414. 10.1016/j.isci.2022.105414 36388963 PMC9646950

